# Viola: a structural variant signature extractor with user-defined classifications

**DOI:** 10.1093/bioinformatics/btab662

**Published:** 2021-09-17

**Authors:** Itsuki Sugita, Shohei Matsuyama, Hiroki Dobashi, Daisuke Komura, Shumpei Ishikawa

**Affiliations:** Department of Preventive Medicine, Graduate School of Medicine, The University of Tokyo, Bunkyo-ku, Tokyo 1130033, Japan; Faculty of Medicine, Tokyo Medical and Dental University, Bunkyo-ku, Tokyo 1138510, Japan; Faculty of Medicine, Tokyo Medical and Dental University, Bunkyo-ku, Tokyo 1138510, Japan; Faculty of Medicine, Tokyo Medical and Dental University, Bunkyo-ku, Tokyo 1138510, Japan; Department of Preventive Medicine, Graduate School of Medicine, The University of Tokyo, Bunkyo-ku, Tokyo 1130033, Japan; Department of Preventive Medicine, Graduate School of Medicine, The University of Tokyo, Bunkyo-ku, Tokyo 1130033, Japan

## Abstract

**Summary:**

Here, we present Viola, a Python package that provides structural variant (SV; large scale genome DNA variations that can result in disease, e.g. cancer) signature analytical functions and utilities for custom SV classification, merging multi-SV-caller output files and SV annotation. We demonstrate that Viola can extract biologically meaningful SV signatures from publicly available SV data for cancer and we evaluate the computational time necessary for annotation of the data.

**Availability and implementation:**

Viola is available on pip (https://pypi.org/project/Viola-SV/) and the source code is on GitHub (https://github.com/dermasugita/Viola-SV).

**Supplementary information:**

Supplementary data are available at *Bioinformatics* online.

## 1 Introduction

Somatic mutations in cancer are the cumulative result of DNA aberrations caused by diverse mutational processes. Recently, large scale studies of human cancer have revealed characteristic patterns of mutation types, i.e. mutational signatures, arising from specific processes of single nucleotide variant formation. These studies often provide theoretical explanations for known mutational processes and their consequences, e.g. C>A substitutions and CC>TT alterations caused by smoking and ultraviolet light exposure, respectively.

Structural variants (SVs) are another type of DNA mutation, defined as events larger than 50 bp in size or involving multiple chromosomes, occupying non-negligible proportions of mutations in cancer cells (Mills *et al.*, 2011; Yi and Ju, 2018 ). Signature analysis of SVs may potentially provide novel insights into carcinogenesis. The development of high-throughput sequencing technologies and powerful SV callers has improved the accuracy of SV event identification. Several mechanisms of SV formation have also been identified (Yi and Ju, 2018). Therefore, research on SV signatures is gradually becoming realistic.

To date, several attempts have been made to decompose SV patterns into SV signatures, but an established method has yet to be realized. Previous studies have mainly classified SVs according to segment size and revealed an association between small tandem duplications and BRCA1 mutations (Li *et al.*, 2020; Nik-Zainal *et al.*, 2016). However, a consensus has not been achieved on a precise SV classification method.

SVs can be classified by metrics other than length. Li *et al.* (2020) also used replication timing and common fragile sites (CFSs). Interestingly, the biological meaningfulness of replication timing and CFSs has been reported, e.g. the signatures of medium-sized (50–500 kb) tandem duplications occurring at the site of late replication timing have been associated with CDK12 driver mutations, whereas CFS signatures have been associated with gastrointestinal cancer. Other SV classification methods, such as microhomology and association of transposons, have yet to be considered in detail; therefore, further analysis is required to identify a suitable SV classification method for signature analysis.

At present, very few tools are available for SV signature analysis. To the best of our knowledge, pyCancerSig (Thutkawkorapin et al., 2020), which is the first tool that can handle SVs for cancer mutation signature analysis, is the only SV signature analysis tool currently available. However, pyCancerSig has limitations in SV classifications as it only supports traditional SV classes, i.e. deletion, duplication, inversion and translocation, and length-based classification.

The time-consuming nature of parsing variant call format (VCF) files is also an obstacle to SV analysis. VCF is the *de facto* standard format by which genetic variant data are recorded with high human readability. However, from a data management perspective, VCF can be a bottleneck for analysis owing to its complex structure. For SVs in particular, accurate interpretation of VCF records at the single nucleotide level requires considerable learning costs. Difficulties with VCF interpretation cannot be ignored because even 1 bp error in positioning SVs can have critical consequences, e.g. in microhomology analysis.

Merging SV calls from different callers is also an issue in SV analysis. Precision of SV detection can be improved by merging the results of multiple SV callers (Cameron *et al.*, 2019; Kuzniar *et al.*, 2020); however, different SV callers use different ways to represent VCF files, which makes integration challenging.

Here, we present Viola, a highly customizable and flexible Python package that supports SV signature analysis with user-defined SV classification, matrix-generation functions, and a file exportation system that is compatible with external statistical utilities and facilitates interpretation of results. Viola accepts VCF files from four popular SV callers, namely Manta, Delly, Lumpy and Gridss, and can also read BEDPE format (Cameron et al., 2017; Chen *et al.*, 2016; Layer *et al.*, 2014; Rausch *et al.*, 2012). Viola also provides an intuitive VCF file manager for filtering, annotating, converting VCF to BEDPE and multicaller merging.

## 2 Implementation

### 2.1 Data structure

Viola converts input SV data files, such as VCF and BEDPE files, into our original Python classes. Instances of these classes store SV data as a set of tidy rectangular tables linked via identifiers such as SV ID output by the SV callers (Supplementary Fig. S1). These tables follow the principles of tidy data, i.e. each SV record is a row, each variable is a column and each type of observational unit is a table (Wickham, 2014). Consequently, storage of multiple values in one element is avoided, in contrast to the INFO and FORMAT columns of a VCF file. Hence, a specific single value can be accessed by simply specifying the row and column of the table of interest; this provides freedom in data handling without the need for cumbersome codes.

### 2.2 User interface

Viola is written in the Python programming language. Although it is intended for use within Python scripts, some features are available from the command line.

**Fig. 1.**
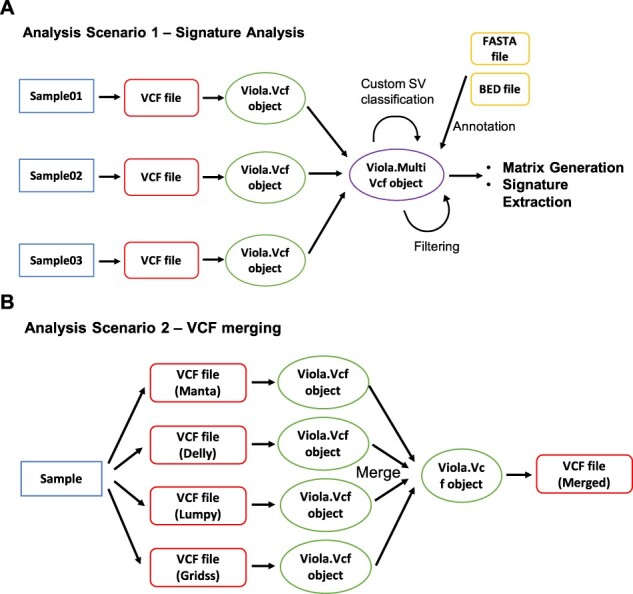
Visualization of the data flow in the main analysis scenarios. (**A**) Process of feature matrix generation from multiple samples. (**B**) Overview of VCF merging system

Viola supports SV signature analysis with user-defined SV classes (Fig. 1A and Supplementary Fig. S1A and B). A simple feature matrix based on traditional SV types and SV length, output by the SV caller can be generated from the command line. Advanced uses such as annotation, filtering and multicaller intersection, which are required to generate a complex feature matrix, are supported within Python scripts. In combination with these functions, it is possible to define a wide variety of SV classes, such as ‘duplications located on CFS sites’ and ‘deletions <50 kb in size, located on the early replication timing zones’. These operations can be implemented with simple syntax and are designed to refine the SV classification by trial and error (Supplementary Fig. S2B).

From an internal data structure perspective, user-defined SV classes are interpreted as new INFO entries of the VCF file. Hence, users can output new VCF or BEDPE files with annotation of novel SV classes as well as generating a signature-analysis-ready feature matrix according to these additional SV classes.

Alongside signature analysis, Viola has the following features:

- Support of well-known SV callers including Manta, Delly, Lumpy and Gridss. The notation has been unified as much as possible to facilitate subsequent processing including merging (Fig. 1B).
- Fast annotation methods that utilize the interval tree algorithm. Source files in BED format are acceptable; thus, information such as gene names, CFSs, replication timing and copy number can be annotated if they can be expressed in BED format.
- An intuitive method for filtering SV records. In addition to filtering for genomic coordinates and INFO fields, filtering for FORMAT fields is possible.
- Estimations of the length and sequence of microhomology from SV breakpoint positions. Where SV callers do not return microhomology information or publicly available SV data does not contain such information, Viola can estimate microhomology using the reference sequence.

The use of these characteristics is described in detail in the official Viola documentation, which is available online (https://dermasugita.github.io/ViolaDocs/docs/html/index.html).

### 2.3 Custom SV classification overview

With Viola, any information in the INFO field of the VCF can be used for SV classification. Many SV callers write the SV type and length in the INFO field by default making it easy to classify by these variables. For BEDPE files that do not define a field corresponding to the INFO field in a VCF file, Viola will automatically generate INFO fields such as SV length and type. Additionally, new INFO fields can be added using BED file annotation and microhomology prediction. BED files can be used to annotate genes, CFSs, replication timing, copy numbers, etc., which individually or in combination can be used to classify SVs.

For usability, two SV classifications are available as default settings of the Viola function. One is a simple length-based classification, and the other is the same classification as the analysis in Section 2.5 (Supplementary Table S1A and B).

### 2.4 VCF merging strategy

Viola provides multicaller merging systems. SVs from the different callers will be merged into a single SV when the following conditions are satisfied: (i) the genomic coordinates of multiple SV break-ends are close to each other based on the user-specified criteria (proximity-based or confidence interval-based criteria as described below). (ii) The strands of the SV break-ends are concordant. (iii) The SVs overlap each other at least 1 bp. The latter two conditions are included to avoid merging discordant SV types and small non-overlapping SVs.

Currently, two criteria for genomic coordinates evaluation have been implemented: proximity-based and confidence interval-based criteria. The former uses the representative genomic coordinates, e.g. POS field and END entry of INFO field. Multiple SV records within a user-defined threshold will be merged. The latter employs confidence intervals reported by SV callers on the CIPOS and CIEND entries of the INFO field. The multiple SV records will be considered a single event when their confidence intervals share at least 1 bp of genomic coordinates.

### 2.5 Application 

#### 2.5.1 Matrix generation with simple code

We ran Viola to generate an SV feature matrix using public BEDPE files reported in a PCAWG study (Li *et al.*, 2020). First, we downloaded 2748 BEDPE files from the ICGC data portal (https://dcc.icgc.org/releases/PCAWG/consensus_sv) and used Viola to read 2605 of these files that were not empty as a MultiBedpe instance. Second, the instance was successfully annotated by CFSs and replication timing BED files that we built according to the PCAWG study. We defined 25 SV classes according to CFSs, replication timing and SV length and then generated a 2605 × 25 feature matrix. These operations were written in only 11 lines of the Python code, excluding code for custom SV definitions (Supplementary Fig. S2A). The matrix generated here can be easily reproduced by following the tutorial in the Viola official document.

#### 2.5.2 Signature extraction analysis

We extracted nine SV signatures from the generated matrix using a function of Viola that simultaneously performs non-negative matrix factorization and cluster stability evaluation (Supplementary Figs S3 and S4). Several signatures, including the signatures of CFSs, small deletions (<50 kb) and small duplications (<50 kb), were comparable to those in the PCAWG study (Li *et al.*, 2020). We further explored the association between each of the nine signatures and driver mutations of three well-known DNA repair genes: *BRCA1*, *BRCA2* and *CDK12* (Supplementary Table S2). These genes were significantly associated with the small duplication signature, small deletion signature and medium–large duplication signature, as expected from previous studies (Li *et al.*, 2020; Menghi *et al.*, 2018; Nik-Zainal *et al.*, 2016; Popova *et al.*, 2016) (Supplementary Table S1).

#### 2.5.3 Multicaller VCF merging

We synthesized VCF files mimicking outputs from Manta, Delly, Lumpy and Gridss. These files shared several SVs recorded with errors within 100 bp. In addition, they were designed as the confidence intervals of shared SV break-ends overlapped each other. Four VCF files were merged by Viola with two methods, proximity-based and confidence interval-based criteria, which the user can select.

First, we tested proximity-based merging with 100 bp specified as the option for proximity. SV events located within 100 bp were given the same ID. We removed SV records called by only a single SV caller. All shared SVs were merged as expected and successfully exported as a VCF file (Supplementary Data S1).

Second, we examined confidence interval-based merging. When SV events that their confidence intervals shared the genomic coordinates at least 1 bp, they were merged and given the same ID. SV records supported by a single SV caller were filtered out. The obtained VCF file was the same as expected.

#### 2.5.4 Annotation performance

We tested the performance of the annotations on 2605 BEDPE files using 18 lines of CFS BED files. In total, 618 492 break-ends were annotated according to whether each was present or absent on the CFS. On average, this took 7.5 min to complete using a single thread on an Ubuntu x86_64 server (Intel Core i7-8700K CPU at 3.70 GHz).

## 3 Future works

Although Viola provides useful functions for SV data manipulation, especially SV signature analysis, further enhancements would make the software more meaningful, covering a wider range of research questions. First, functions for handling more complex SV events, such as chromothripsis and chromoplexy that result from multiple DNA damage occurring in a single event, could be desirable (Li *et al.*, 2020; Stephens *et al.*, 2011). Such features may lead to the discovery of new SV signatures and the elucidation of the mechanistic basis of SV formation. Second, a more detailed annotation system would facilitate a more specific characterization of each SV event, because the current version of Viola does not support annotation with nucleotide sequence-level analysis, e.g. the frameshift status of affected genes or the impact of putative fusion genes, with the exception of microhomology inference. Such a detailed annotation system would facilitate a more specific characterization of each SV event. Finally, more types of SV callers need to be supported including those for long-read sequencing technology.

## 4 Conclusion

We developed Viola, a tool for SV signature analysis that allows highly customizable SV classification. This tool also overcomes the difficulty of parsing current VCF files as well as the problem of different notations derived from different callers. Viola will help stimulate cancer genome research to better understand the biological significance of SVs.

## Supplementary Material

btab662_Supplementary_Data
