# Supplementary material for: Viola: a structural variant signature extractor with user-defined classifications
Source: Bioinformatics. 2021 Sep 17;38(2):540–2. doi: 10.1093/bioinformatics/btab662 (PMC8723148; doi:10.1093/bioinformatics/btab662)
Supplement: btab662_Supplementary_Data [file btab662_supplementary_data.zip › revised_supplementary_pubr.docx]

**Supplementary Information**

**Data Structure of Viola’s Objects**

Viola breaks down the SV information into multiple tidy tables to enable flexible data processing. The most important benefit of this data structure is the extendibility for future functions. Software always requires updates to keep up with trends and developments. By breaking down the SV information into multiple tidy tables, even complex requirements can be implemented with little effort.

Supplementary Figure S1. explains the specific data structure of Viola’s Vcf class.

**Supplementary Figure S1.** **Data structure of a viola.Vcf object.** The upper part of the figure shows an example of a Manta-like VCF. As shown in the lower part of the figure, the viola.Vcf object holds the information of a VCF file in several rectangular tables. The tables are related to each other by VCF IDs. The grey columns are the primary key or composite primary key of the table. The header information of the VCF is also stored as tables (not shown). Abbreviations: POS: start position of the SV; END: end position of the SV; SVLEN: length of the SV; SVTYPE: type of SV; CIPOS: confidence interval around POS; CIEND: confidence interval around END; MATEID: ID of mate break end; SU: count of supporting reads of the SV; PR: count of paired end reads supporting the SV; SR: count of split reads supporting the SV.

**Supplementary Figure S2.** **Example code for feature matrix generation**. **(A)** (1) Import Viola package. (2) Read BEDPE files under the “pcawg” directory as viola.MultiBedpe object. (3 and 4) Load common fragile site and replication timing BED/BEDGRAPH for annotation*. (5 and 6) Annotate “pcawg_bedpe” variable with the BED/BEDGRAPH loaded above. (7) Obtain mean replication timing for each SV breakpoint. (8–11) Classify custom SV type according to the definition file and export feature matrix. **(B)** Definition file for custom SV classification. Each SV class definition consists of a line specifying the SV class name, lines describing the conditions, and a line passing the set operation of the conditions. Note that the file content shown here is part of all SV definitions used in this study. **(C)** File tree of this analysis.

* Currently, a clear distinction between BED and BEDGRAPH files is not made in relation to the annotation of Viola objects since only the first four columns of these files are used for annotation purposes.

**Supplementary Table S1A. Simple length-based SV classification supported by Viola.**

| **Simple length-based classification** |
| --- |
| Deletion shorter than 50 kb |
| Deletion shorter than 500 kb |
| Deletion shorter than 5 Mb |
| Deletion longer than 5 Mb |
| Duplication shorter than 50 kb |
| Duplication shorter than 500 kb |
| Duplication shorter than 5 Mb |
| Duplication longer than 5 Mb |
| Translocation |
| Inversion shorter than 100 kb |
| Inversion longer than 100 kb |

**Supplementary Table S1B. SV classification used in the analysis of “Application” section of this article.**

| **SV classification used in the "Application" section** |
| --- |
| Deletion at fragile site |
| Duplication at fragile site |
| Deletion shorter than 50 kb in the region of early replication timing |
| Deletion shorter than 50 kb in the region of middle replication timing |
| Deletion shorter than 50 kb in the region of late replication timing |
| Deletion shorter than 500 kb in the region of early replication timing |
| Deletion shorter than 500 kb in the region of middle replication timing |
| Deletion shorter than 500 kb in the region of late replication timing |
| Deletion shorter than 5 Mb in the region of early replication timing |
| Deletion shorter than 5 Mb in the region of middle replication timing |
| Deletion shorter than 5 Mb in the region of late replication timing |
| Deletion longer than 5 Mb |
| Duplication shorter than 50 kb in the region of early replication timing |
| Duplication shorter than 50 kb in the region of middle replication timing |
| Duplication shorter than 50 kb in the region of late replication timing |
| Duplication shorter than 500 kb in the region of early replication timing |
| Duplication shorter than 500 kb in the region of middle replication timing |
| Duplication shorter than 500 kb in the region of late replication timing |
| Duplication shorter than 5 Mb in the region of early replication timing |
| Duplication shorter than 5 Mb in the region of middle replication timing |
| Duplication shorter than 5 Mb in the region of late replication timing |
| Duplication longer than 5 Mb |
| Translocation |
| Inversion shorter than 100 kb |
| Inversion longer than 100 kb |

**Signature Extraction Procedure**

Here, we describe how SV signatures were extracted from the PCAWG dataset. To determine the number of signatures, *K*, we evaluated the stability of signatures derived from non-negative matrix factorization (NMF) and its reconstruction error. Detailed steps are provided below.

1. Generate a new 2,605 × 25 matrix, $\dot{M}$, by bootstrapping the original matrix M. Here each element $\dot{m}_{i, j}$ of $\dot{M}$ is chosen with a probability of $m_{i, j}/\sum_{i,j} m_{i, j}$, where $m_{i, j}$ is each element of M while $\sum_{i, j} \dot{m}_{i, j}= \sum_{i, j} m_{i, j}$.
2. Apply NMF to the bootstrapped matrix $\dot{M}$ to obtain an exposure matrix, $\dot{E}$, with 2,605 × *K* and a signature matrix, $\dot{P}$, with *K* × 25. $\dot{E}$ and $\dot{P}$ are initialized by a non-negative double singular decomposition method with zeros filled with the average of $\dot{M}$. Kullback–Leibler divergence is used for loss function.
3. Perform step 1 and 2 for 100 iterations to obtain 100K signatures.
4. Use a K-means method for clustering 100K signatures into K clusters with the constraint that signatures from the same iteration should not been assigned to the same cluster. The average silhouette score is calculated for stability evaluation.
5. The average signature matrix $\bar{P}$ is constructed with *K* × 25. Each row of $\bar{P}$ is the centroid of the K-means clustering performed in step 4. The average exposure matrix $\bar{E}$ is then calculated by NMF using the original matrix M and $\bar{P}$, where the matrix $\bar{P}$ is not updated while NMF. Finally, the Kullback–Leibler divergence of M and $\bar{E} \times\bar{P}$ was calculated as reconstruction error.

Steps 1–5 were conducted for each *K* ranging from 2 to 13 (Supplementary Figure S3).

**Supplementary Figure S3.** **Average silhouette score of K-means clusters and reconstruction error for the number of signatures (K).** After a manual assessment of each K with reference to the stability score and reconstruction error, we chose K = 9 as the number of signatures. Extracted signatures are shown in Supplementary Figure S4.

**Supplementary Figure S4.** **Nine signatures extracted from the PCAWG dataset using Viola.**

**Statistical testing of the association between signatures and driver mutations**

We obtained several signatures that were comparable with those in the PCAWG report such as the small deletion signature and medium-large duplication signature. Statistical significance was tested for the effect of driver mutations in BRCA1, BRCA2, and CDK12 on the nine signatures. The p-value of each signature was calculated using a linear model that considered the histological type of each sample (Supplementary Table S1). Specifically, the presence or absence of each driver mutation was set as the objective variable, and the respective SV signature and one-hot-encoded histological type were used as explanatory variables. The R language was used for statistical testing.

**Supplementary Table S2**. **Statistical significance of the effect of driver mutations in *BRCA1*, *BRCA2*, and *CDK12* on nine signatures.** Negative log *p* values are shown (**p* < 0.01, two-tailed t-test).

|  | ***BRCA1*** | ***BRCA2*** | ***CDK12*** |
| --- | --- | --- | --- |
| **Fragile Site** | 0.205 | 0.527 | 0.192 |
| **Small Deletion** | 0.084 | **23.278^*^** | 0.750 |
| **Medium-Large Deletion** | 0.401 | 1.142 | 0.036 |
| **Small Duplication** | **26.030^*^** | 1.579 | 1.055 |
| **Medium Early Duplication** | 0.389 | 0.421 | 0.057 |
| **Medium-Large Duplication** | 0.663 | 1.526 | **6.251^*^** |
| **Translocation** | 0.122 | 0.218 | 0.950 |
| **Small Inversion** | 0.729 | 0.520 | 0.128 |
| **Large Events** | **2.877^*^** | **2.042^*^** | 0.479 |
